# Author Correction: Placental growth factor exerts a dual function for cardiomyogenesis and vasculogenesis during heart development

**DOI:** 10.1038/s41467-023-44507-1

**Published:** 2024-01-04

**Authors:** Nevin Witman, Chikai Zhou, Timm Häneke, Yao Xiao, Xiaoting Huang, Eduarde Rohner, Jesper Sohlmér, Niels Grote Beverborg, Miia L. Lehtinen, Kenneth R. Chien, Makoto Sahara

**Affiliations:** 1 Department of Cell and Molecular Biology, Karolinska Institutet, A6 Biomedicum, SE-171 77 Stockholm, Sweden; 2 Department of Cardiology, University Medical Center Groningen, University of Groningen, Groningen, The Netherlands; 3 Department of Cardiac Surgery, Heart and Lung Center, Helsinki University Hospital and University of Helsinki, Helsinki, Finland; 4 Department of Surgery, Yale University School of Medicine, 333 Cedar Street, New Haven, CN 06510 USA

**Keywords:** Cell lineage, Heart stem cells, Stem-cell differentiation, Data processing

Correction to: *Nature Communications* 10.1038/s41467-023-41305-7, published online 05 September 2023

The original version of this Article omitted from the author list the 9th author, Miia L. Lehtinen, who is from the Department of Cell and Molecular Biology, Karolinska Institutet and Department of Cardiac Surgery, Heart and Lung Center, Helsinki University Hospital and University of Helsinki, Helsinki, Finland.

The corrected version of the Acknowledgements removes the following from the original version: ‘Miia Lehtinen.’

Additionally, the following was added to the Author Contributions: ‘M.L.L. performed in vivo experiments relating to the primate works.’

This has been corrected in both the PDF and HTML versions of the Article.

