# Supplementary material for: Placental growth factor exerts a dual function for cardiomyogenesis and vasculogenesis during heart development
Source: Nat Commun. 2023 Sep 5;14:5435. doi: 10.1038/s41467-023-41305-7 (PMC10480216; doi:10.1038/s41467-023-41305-7)
Supplement: Supplementary file 1 — Supplementary Information [file 41467_2023_41305_MOESM1_ESM.pdf]

Supplementary Information

Supplementary Tables and Figures

Supplementary Table 1. Primary antibodies used for flow cytometry, immunostaining, western blotting and ChIP experiments.

Primary Antibody	Clone	Purpose	Company	Catalog Number	Dilution
α-SMA	1A4	Immunostaining	Sigma-Aldrich	A2547	1:200
β-actin-HRP		Western blotting	Cell Signaling Technology	5125S	1:2000
CD34-APC	581	Flow cytometry	BD Biosciences	555824	1:100
EOMES	EPR21950-241	ChIP*	Abcam	ab216870	1:50
EOMES	polyclonal	Western blotting	Abcam	ab23345	1:1000
ISL1	39.4D5	Immunostaining	DSHB	39.4D5	1:20
ISL1-PE	Q11-465	Flow cytometry	BD Biosciences	562547	1:100
Ki67	B56	Immunostaining	BD Bioscience	550609	1:25
Ki67-FITC	B56	Flow cytometry	BD Biosciences	556026	1:20
MF20		Immunostaining	DSHB	P13538	1:10
MLC2V	polyclonal	Immunostaining	Proteintech	10906-1-AP	1:100
PDGFRB-PE	28D4	Flow cytometry	BD Biosciences	558821	1:100
PECAM1	89C2	Immunostaining	Cell Signaling Technology	3528	1:50
PECAM1-AF647	M89D3	Flow cytometry	BD Biosciences	558094	1:100
PLGF	polyclonal	Immunostaining<br>Western blotting	Abcam	ab9542	1:100<br>1:500
SM22	polyclonal	Flow cytometry<br>Immunostaining	Abcam	10135	1:100
SOX17	EPR20684	Western blotting<br>ChIP*	Abcam	ab224637	1:500<br>1:30
TNNT2	13-11	Immunostaining	Thermo Fisher Scientific	MS-295-P1	1:50
TNNT2	EPR3695	Immunostaining	Abcam	ab91605	1:50
TNNT2-APC	REA400	Flow cytometry	Miltenyi Biotec	MB-130-120-403	1:100
VE-cadherin	polyclonal	Immunostaining	R&D	AF938	1:50
VE-cadherin-PE	55-7H1	Flow cytometry	BD Biosciences	560410	1:100
Vimentin	polyclonal	Immunostaining	Millipore	AB5733	1:1000

*ChIP, chromatin immunoprecipitation

Supplementary Table 2. PCR primers for detection of the putative EOMES- or SOX17-binding sites at the promoter region of the human PLGF gene.

**EOMES-target region**	**Target Sequence**	**Location***	**Forward primer**	**Reverse primer**
Region 1	CTCACACCTG	−1934	CAGGCAATACATGCCAAATG	CTCAAGTGATCCACCCACCT
Region 2	CTCACACA	−1408	ACTCACCCTCACACACATGC	TCCTGACGAGCTTCAAACAA
Region 3	ATCACACA<br>GTCACACG	−792<br>& −724	ATACACAGCGGACACGAACA	CTGAGCCTCTGTGTGGTCTG
Negative control	–	−540	GCAGCGTACAGTTCCTCCTC	CTCCCACAGGAAACCTCTCA
**SOX17-target region**	**Target Sequence**	**Location***	**Forward primer**	**Reverse primer**
Region 1	GCATTGTCA	−3901	GCAGACTGGAAGCCATGA	GAGTCGGGCAGATACAGATAAA
Region 2	ACACAATAG	−1638	AGAGCAAGACTCTGTCTCAGAA	GCGCGTGTGCTTAAGTATCT
Region 3	GCACAATAC	−1542	CATCCCATGCATAAGTGCTAGA	TGGGCACAGGTGTGTATTG
Region 4	CCACAATGA	−1384	TGCACAAACACACACCTACTC	CATCCTGACGAGCTTCAAACA
Negative control	–	−400	TGGTAGGTCGGGTTAGAGTT	GGAAAGTGTGTGTGTGTTGTG

*Location of the target regions from the transcription start site (TSS).

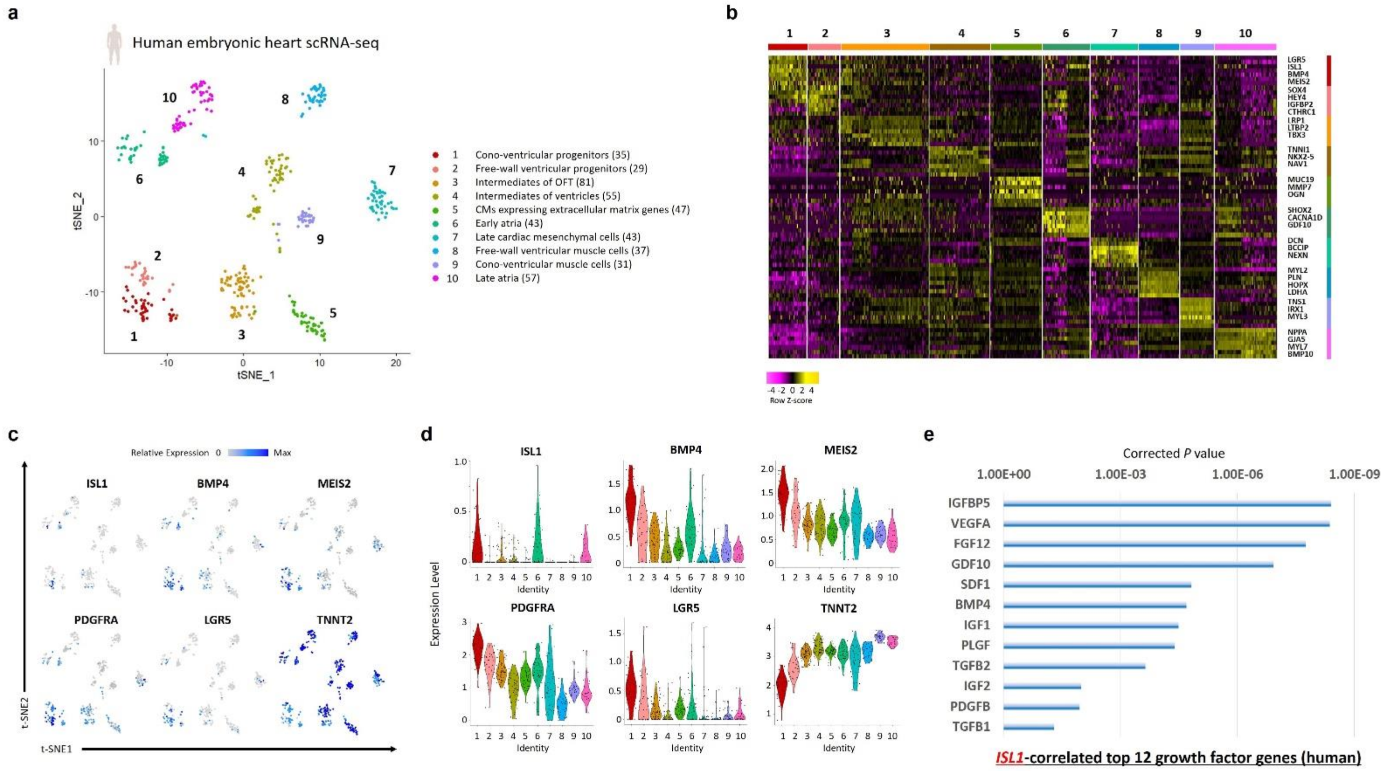

Supplementary Figure 1. Single-cell RNA-seq analysis of human embryonic hearts.

a, The tSNE analysis using the single-cell RNA-seq dataset, which was obtained from human embryonic hearts (4.5 to 10 weeks of fetal ages)[6], segregated 458 individual cardiac cells into 10 molecularly distinct clusters, including a cono-ventricular region-specific heart progenitor (CVP; cluster #1). **b**, Heatmap image showing the representative differentially expressed genes in each of the 10 clusters in **a**. **c**, Feature plots of the early cardiogenic and second heart field (SHF) progenitor-specific genes, which the CVP specifically expressed, as well as a pan-CM marker *TNNT2* on the tSNE plots in **a**. **d**, Violin plots of the same genes as in **c**, in the segregated 10 clusters of the human embryonic heart-derived single cells. **e**, The top 12 growth factor genes correlated with expression of the CVP-specific gene *ISL1* in single-cell RNA-seq data of human embryonic hearts. Corrected *P* value for each gene was calculated by Guilt-by-Association and correlation analysis[26]. Source data are provided as a Source Data file.

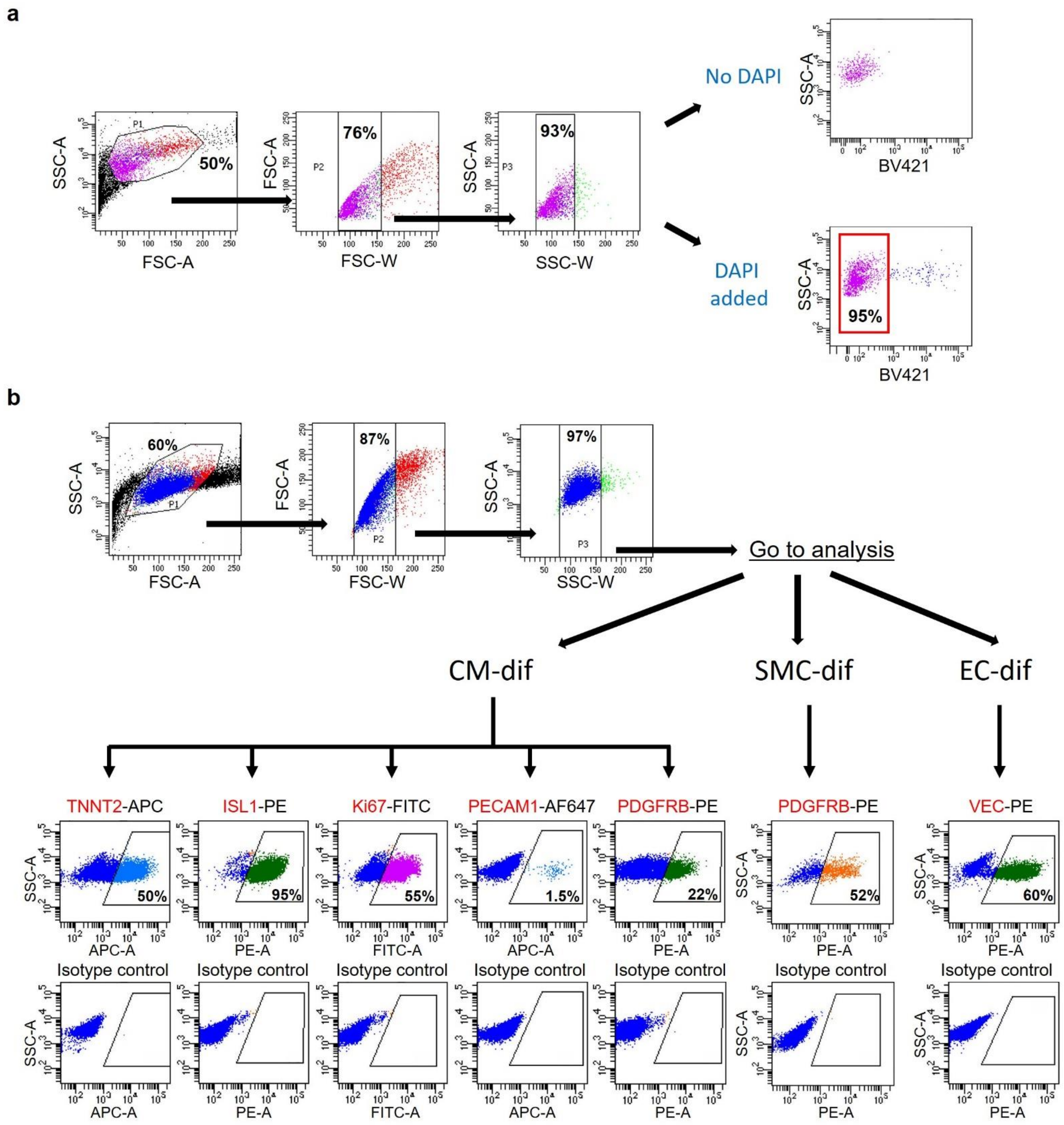

Supplementary Figure 2. Gating strategies on flow cytometry for sorting live cells and analyses.
a, The first gating of primate embryonic heart-derived single cardiac cells was conducted on the FSC and SSC plots to discard cell debris and to obtain intact cells. Then, the gating was on the FSC-W and FSC-A and on SSC-W and SSC-A to specifically discard doublets. The dead cells were stained with DAPI. BV, brilliant violet; FSC, forward scatter; SSC, side scatter. **b**, Gating of TNNT2^{+}, ISL1^{+}, Ki67^{+}, PECAM1^{+}, PDGFRB^{+}, and VE-cadherin (VEC)$^{+}$ cells on flow cytometry analysis during cardiomyocyte (CM), smooth muscle cell (SMC) or endothelial cell (EC) differentiation (dif) is shown. The plots of cells stained with isotype controls are also shown, respectively (bottom). The isotype control antibodies are listed in the Reporting Summary.

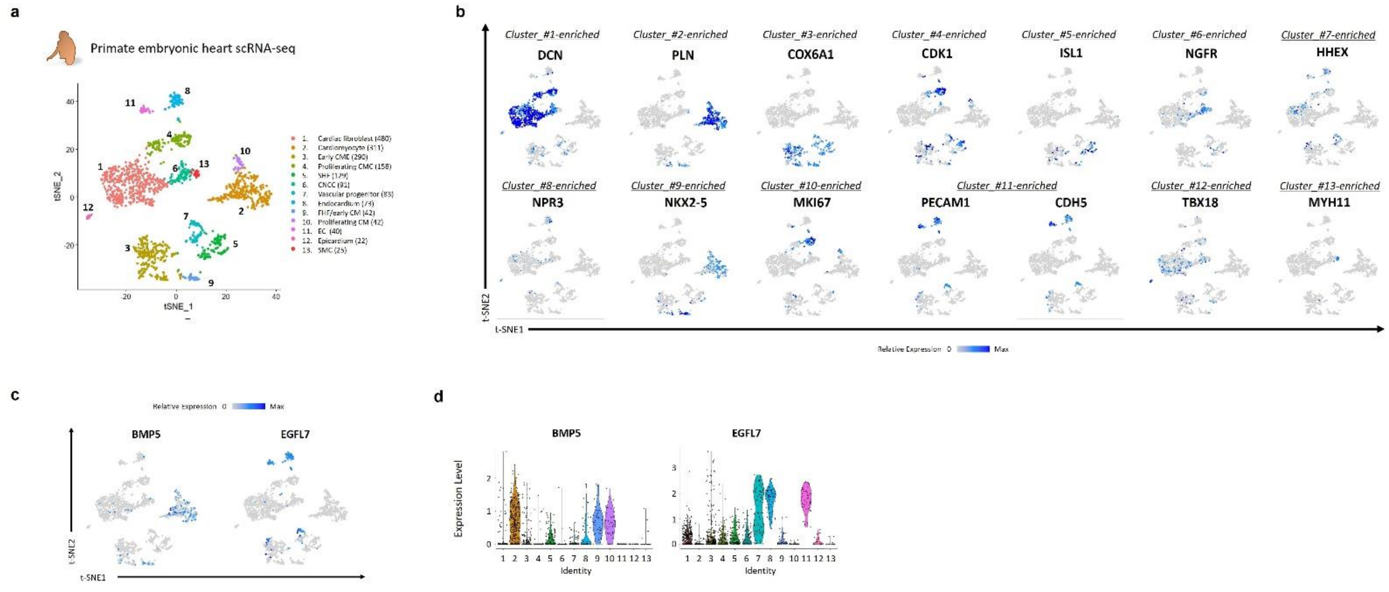

Supplementary Figure 3. Expression patterns of marker genes specific to each of the 13 clusters of the primate embryonic heart-derived single cells.

a, The 13 clusters segregated by the Seurat/tSNE analysis using a total of 1,786 single cardiac cells of primate embryonic hearts. **b**, Feature plots of genes specific to each of the 13 clusters on the tSNE plots in **a**. **c**, Feature plots of bone morphogenetic protein 5 (BMP5; left) and epidermal growth factor-like protein 7 (EGFL7; right) on the tSNE plots in **a**. **d**, Violin plots of BMP5 (left) and EGFL7 (right) in the segregated 13 clusters of the primate embryonic heart-derived single cells.

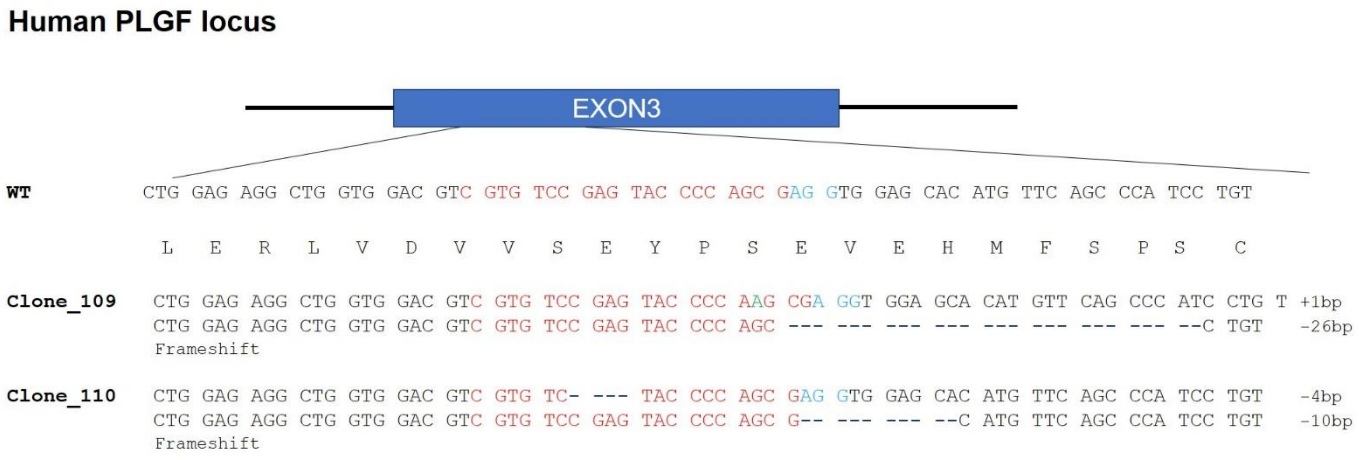

Supplementary Figure 4. Generation of the *PLGF*-KO hESC lines by CRISPR-Cas9.

The top row shows a wild-type (WT) sequence of the region in the third exon of the human *PLGF* gene. The red sequence represents the target sequence of the sgRNA (Methods), and the blue sequence represents the protospacer adjacent motif (PAM). Sequences on the middle and bottom rows are highlighting frameshift mutations of the sgRNA-targeted region in both alleles of the two mutated clones (clone number: 109 [middle] and 110 [bottom]). Within the sequences, deletions are indicated by dashed lines, and an insertion is indicated by a green letter.

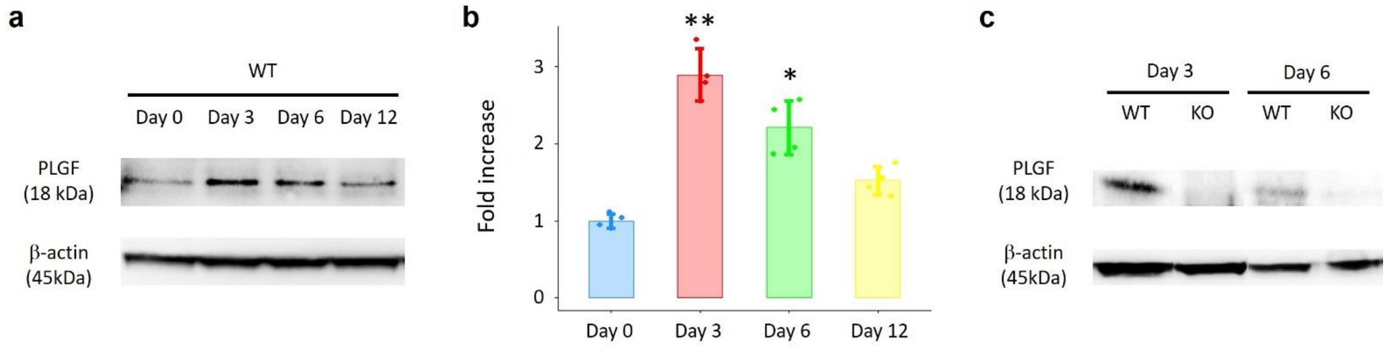

Supplementary Figure 5. Validation of the *PLGF*-KO hESC clone.

a, Western blotting analysis for expression of PLGF protein with β-actin protein (a loading control) in WT cells at days 0, 3, 6 and 12 in hESC-CM differentiation. **b**, Quantitative results in **a.** Data are presented as mean ± SD (n=4 independent experiments). Differences between groups were examined with one-way ANOVA followed by Tukey multiple comparisons test. *P<0.01 and **P<0.0001 vs day 0. Source data are provided as a Source Data file. **c**, Western blotting analysis for expression of PLGF protein with β-actin protein in WT and *PLGF*-KO cells at days 3 and 6 in hESC-CM differentiation.

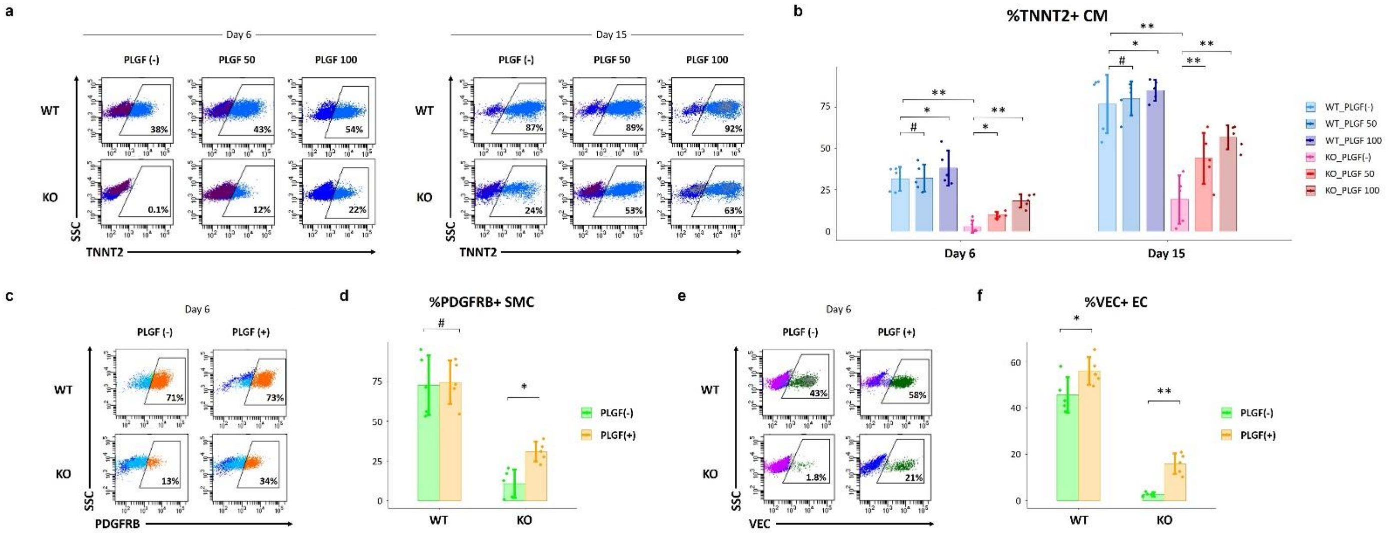

Supplementary Figure 6. Effects of treatment with PLGF protein in *in vitro* hESC differentiation.
a, Representative images on flow cytometry analysis showing the ratios of a differentiated CM marker TNNT2^{+} at days 6 (left) and 15 (right) in WT (top) and *PLGF*-KO (bottom) cells with or without treatment with human recombinant PLGF protein (50 or 100 ng/mL) during days 3-7 in hESC-CM differentiation[18,30]. **b**, Statistical data of the ratios of %TNNT2^{+} in **a**. Treatment with PLGF protein increased %TNNT2^{+} in WT and *PLGF*-KO cells in a dose-dependent manner at days 6 and 15 in hESC-CM differentiation, respectively. **c**, Representative images on flow cytometry analysis showing the ratios of a SMC marker PDGFRB^{+} at day 6 in WT (top) and *PLGF*-KO (bottom) cells with or without treatment with human recombinant PLGF protein (50 ng/mL) during days 4-6 in hESC-SMC differentiation[33]. **d**, Statistical data of the ratios of %PDGFRB^{+} in **c**. Treatment with PLGF protein (50 ng/mL) increased %PDGFRB^{+} in *PLGF*-KO cells at day 6 in hESC-SMC differentiation. **e**, Representative images on flow cytometry analysis showing the ratios of an EC marker VE-cadherin (VEC)$^{+}$ at day 6 in WT (top) and *PLGF*-KO (bottom) cells with or without treatment with human recombinant PLGF protein (50 ng/mL) during days 4-6 in hESC-EC differentiation[34]. **f**, Statistical data of the ratios of %VEC^{+} in **e**. Treatment with PLGF protein (50 ng/mL) increased %VEC^{+} in both WT and *PLGF*-KO cells at day 6 in hESC-EC differentiation. Data in (**b**), (**d**) and (**f**) are presented as mean ± SD (n=5 independent experiments). Differences between groups were examined with one-way ANOVA followed by Tukey multiple comparisons test. $^{\#}P$=not significant, *P<0.05 and **P<0.01. Source data are provided as a Source Data file.

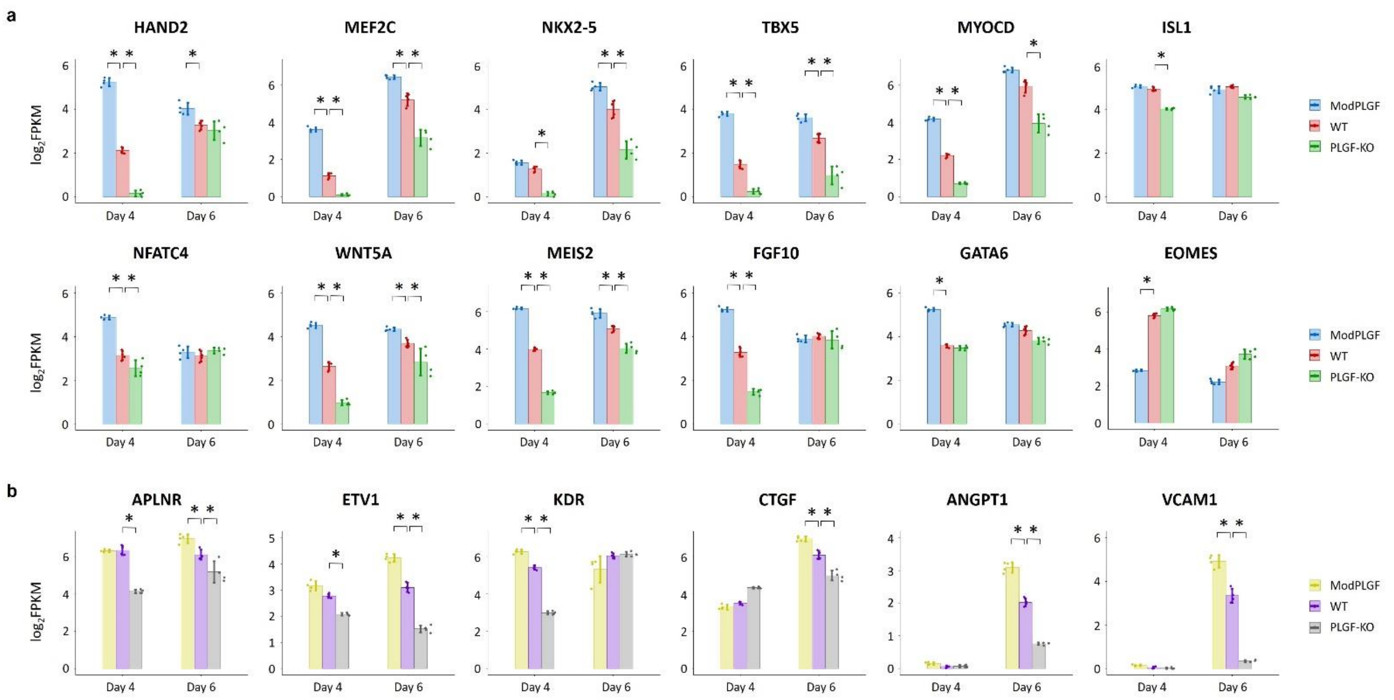

Supplementary Figure 7. Upregulation or downregulation of the cardiomyogenesis and vasculogenesis driver genes by PLGF modRNA transfection or *PLGF* gene deletion.

a, Comparisons of the cardiomyogenesis driver genes among the PLGF modRNA (ModPLGF)-transfected, WT and *PLGF*-KO cells at days 4 and 6 in hESC-CM differentiation. **b**, Comparisons of the vasculogenesis driver genes among the ModPLGF-transfected, WT and *PLGF*-KO cells at days 4 and 6 in hESC-CM differentiation. Data in (**a**) and (**b**) are presented as mean ± SD (n=4 independent experiments). Differences between groups were examined with one-way ANOVA followed by Tukey multiple comparisons test. *P<0.05. Source data are provided as a Source Data file.

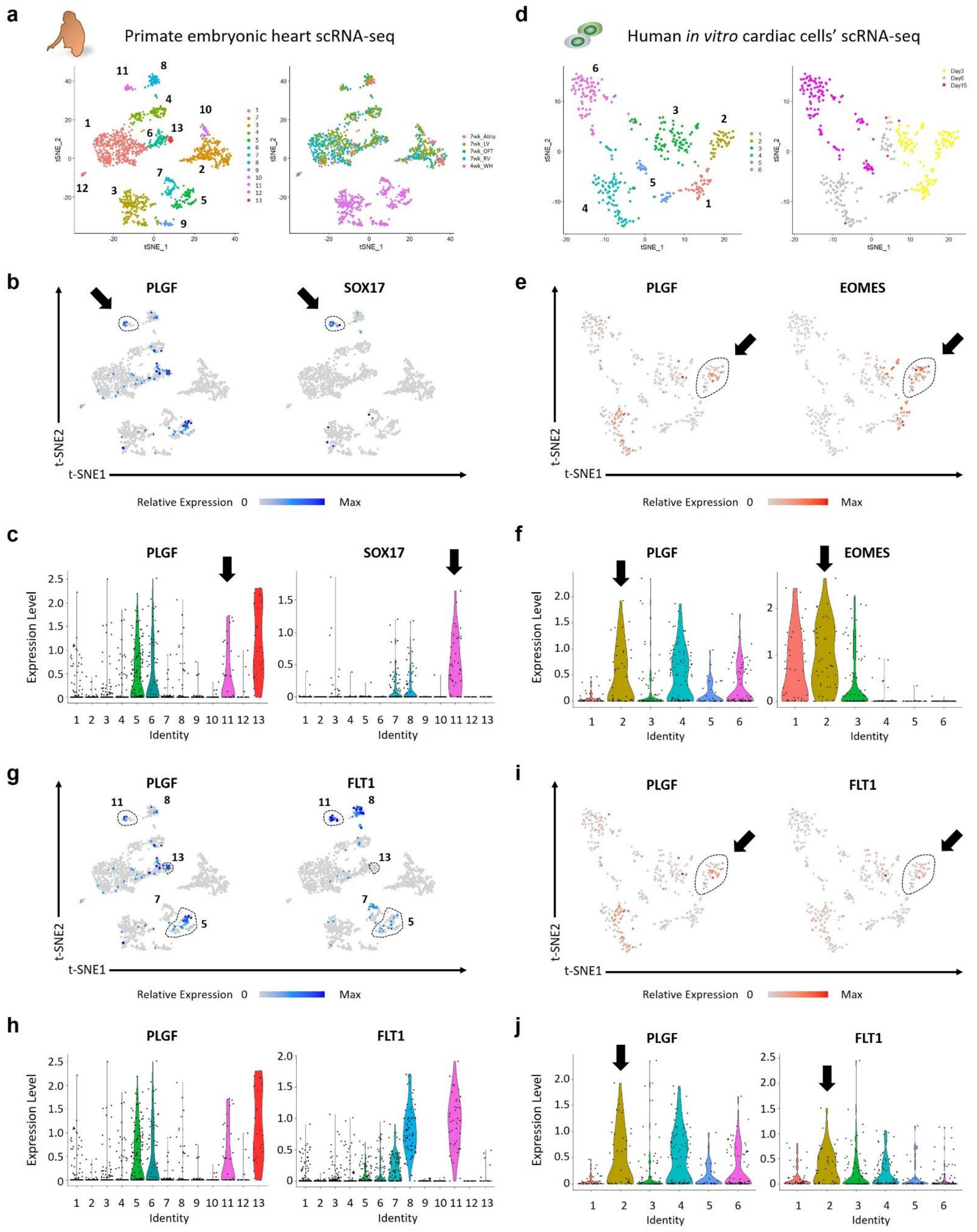

Supplementary Figure 8. Expression patterns of PLGF, SOX17, EOMES, and FLT1 in single-cell RNA-

seq datasets of primate embryonic hearts and *in vitro* hESC-derived cardiac cells.

a, The 13 clusters segregated by the Seurat/tSNE analysis using a total of 1,786 single cardiac cells of primate embryonic hearts (left). The right panel shows the stage (i.e., 4 or 7 week [wk]) and the anatomical locations (i.e., atria, left and right ventricle [LV and RV], and outflow tract [OFT]) of the individual cells in the left panel. **b**, Feature plots of *PLGF* and *SOX17* on the tSNE plots in **a**. Arrows pointed the cluster #11 (ECs). **c**, Violin plots of *PLGF* and *SOX17* in the segregated 13 clusters (**a**) of the primate embryonic heart-derived single cells. Arrows pointed the cluster #11 (ECs). **d**, The tSNE analysis using the single-cell RNA-seq dataset of *in vitro* hESC-derived cardiac cells during CM differentiation (day 3 to 15), which was previously obtained[6]. As shown, this segregated 366 individual cells into 6 clusters (left), including the cluster #2 that was occupied by cells on day 3 in CM differentiation. **e**, Feature plots of *PLGF* and *EOMES* on the tSNE plots in **d**. *EOMES* was entirely expressed in cells of the cluster #2 (arrows), which was thereby considered as early cardiac precursors. The majority (≈70%) of cells in the cluster #2 also expressed *PLGF*. **f**, Violin plots of *PLGF* and *EOMES* in the segregated 6 clusters (**d**) of the *in vitro* hESC-derived single cardiac cells. Arrows pointed the cluster #2 (early cardiac precursors). **g**, Feature plots of *PLGF* and *FLT1* on the tSNE plots in **a**. The clusters #7 (vascular progenitors), #8 (endocardium) and #11 (ECs) are highlighted as the *FLT1*-expressing populations. The cluster #5 (SHF) also partially expressed *FLT1*, while the cluster #13 (SMCs) showed little or no expression of *FLT1*. **h**, Violin plots of *PLGF* and *FLT1* in the segregated 13 clusters (**a**) of the primate embryonic heart-derived single cells. **i**, Feature plots of *PLGF* and *FLT1* on the tSNE plots in **d**. Arrows pointed the cluster #2 (early cardiac precursors). **j**, Violin plots of *PLGF* and *FLT1* in the segregated 6 clusters (**d**) of the *in vitro* hESC-derived single cardiac cells. The early cardiac precursors (cluster #2; arrows) often co-expressed *PLGF* and *FLT1* (**i**,**j**).

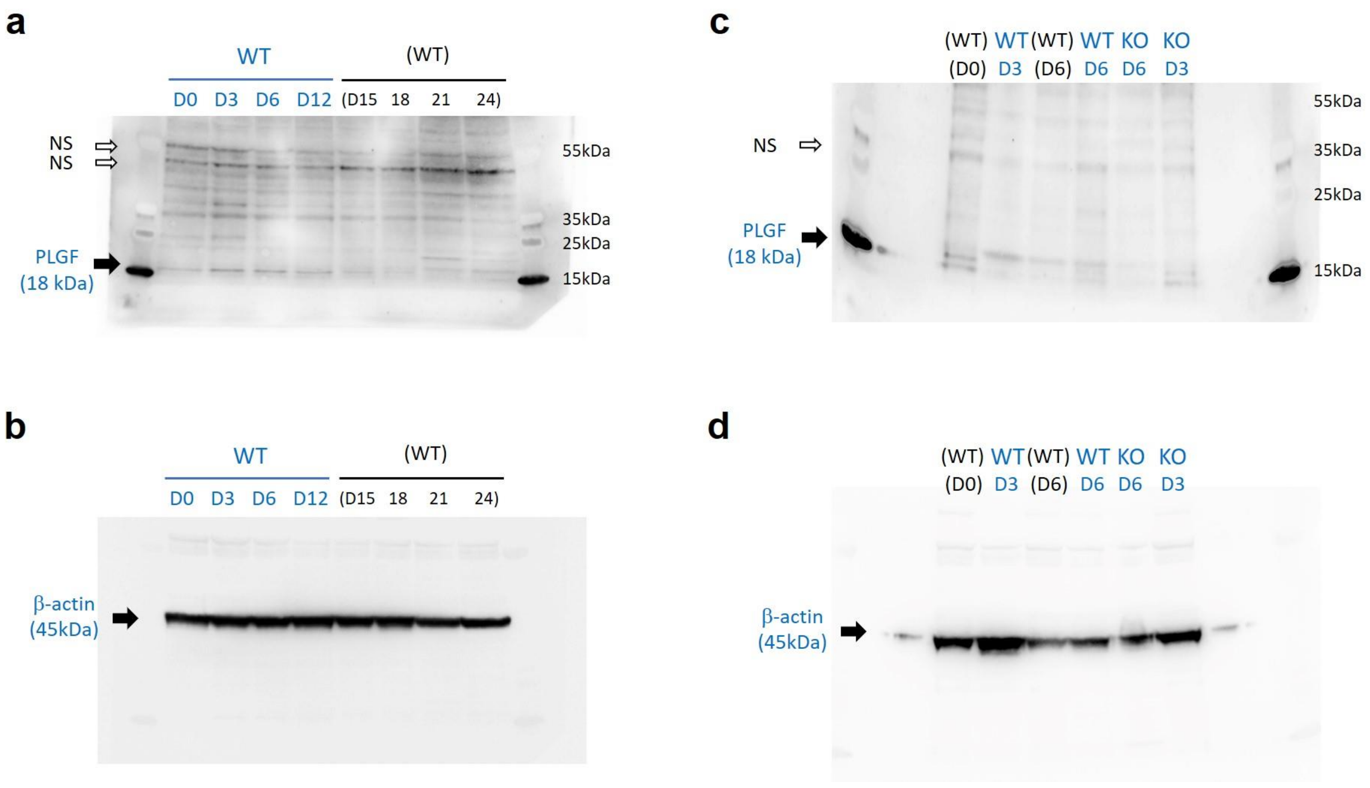

Supplementary Figure 9. Images of the complete unedited gels.

Images of the complete unedited gels for each of the representative cropped gels within the text and figures are shown. **a** is for Supplementary Fig. 3a (top); **b** is for Supplementary Fig. 3a (bottom); **c** is for Supplementary Fig. 3c (top); and **d** is for Supplementary Fig. 3c (bottom). The lanes corresponding with those shown in the cropped gels presented within the text and figures are highlighted in blue color. D, day; KO, knockout; NS, not specific; WT, wildtype.
