# Supplementary material for: Placental growth factor exerts a dual function for cardiomyogenesis and vasculogenesis during heart development
Source: Nat Commun. 2023 Sep 5;14:5435. doi: 10.1038/s41467-023-41305-7 (PMC10480216; doi:10.1038/s41467-023-41305-7)
Supplement: Supplementary file 3 — Description of Additional Supplementary Files [file 41467_2023_41305_MOESM3_ESM.pdf]

Description of Additional Supplementary Files

File Name: Supplementary Data 1

Description: **Representative 52 growth factors analyzed in this study.**

The growth factors indicated by (#) were transcribed into chemically modified mRNA (modRNA) and constituted the unique modRNA library.

File Name: Supplementary Data 2

Description: **List of the modPLGF-transfected, WT and PLGF-KO hESC-derived cardiac differentiating cells-enriched genes at Day 4.**

FC, fold increase; modPLGF, PLGF modified mRNA-transfected cells; PLGF-KO, PLGF-knockout cells; WT, wild-type cells.

File Name: Supplementary Data 3

Description: **List of the modPLGF-transfected, WT and PLGF-KO hESC-derived cardiac differentiating cells-enriched genes at Day 6.**

FC, fold increase; modPLGF, PLGF modified mRNA-transfected cells; PLGF-KO, PLGF-knockout cells; WT, wild-type cells.
