# Supplementary material for: Placental growth factor exerts a dual function for cardiomyogenesis and vasculogenesis during heart development
Source: Nat Commun. 2023 Sep 5;14:5435. doi: 10.1038/s41467-023-41305-7 (PMC10480216; doi:10.1038/s41467-023-41305-7)
Supplement: Supplementary file 7 — Reporting Summary [file 41467_2023_41305_MOESM7_ESM.pdf]

Corresponding author(s): Makoto Sahara

Last updated by author(s): Aug 25, 2023

Reporting Summary

Nature Portfolio wishes to improve the reproducibility of the work that we publish. This form provides structure for consistency and transparency in reporting. For further information on Nature Portfolio policies, see our Editorial Policies and the Editorial Policy Checklist.

Statistics

For all statistical analyses, confirm that the following items are present in the figure legend, table legend, main text, or Methods section.

n/a	Confirmed	
☐	☒	The exact sample size (*n*) for each experimental group/condition, given as a discrete number and unit of measurement
☐	☒	A statement on whether measurements were taken from distinct samples or whether the same sample was measured repeatedly
☐	☒	The statistical test(s) used AND whether they are one- or two-sided<br>*Only common tests should be described solely by name; describe more complex techniques in the Methods section.*
☒	☐	A description of all covariates tested
☐	☒	A description of any assumptions or corrections, such as tests of normality and adjustment for multiple comparisons
☐	☒	A full description of the statistical parameters including central tendency (e.g. means) or other basic estimates (e.g. regression coefficient) AND variation (e.g. standard deviation) or associated estimates of uncertainty (e.g. confidence intervals)
☐	☒	For null hypothesis testing, the test statistic (e.g. *F*, *t*, *r*) with confidence intervals, effect sizes, degrees of freedom and *P* value noted<br>*Give P values as exact values whenever suitable.*
☒	☐	For Bayesian analysis, information on the choice of priors and Markov chain Monte Carlo settings
☒	☐	For hierarchical and complex designs, identification of the appropriate level for tests and full reporting of outcomes
☒	☐	Estimates of effect sizes (e.g. Cohen's *d*, Pearson's *r*), indicating how they were calculated

Our web collection on statistics for biologists contains articles on many of the points above.

Software and code

Policy information about availability of computer code

Data collection

All software used for the data collection is described in detail in the methods section of the manuscript. The following software packages with version numbers were used:

FastQC/0.11.9; cutadapt/1.9.1; samtools/0.1.19; star/2.5.1; htseq/0.9.1;
FACS Diva/v4.1 (Becton Dickinson); Zeiss 710 confocal microscope imaging system (LSM700 and ZEN 2012 SP5 v14.0.23.201);
Jaspar (http://jaspar.genereg.net/); and MatInspector (Genomatix, http://www.genomatix.de/).

Data analysis

All software used for the data analysis is described in detail in the methods section of the manuscript. The following software packages with version numbers were used:

ImageJ/FIJI v1.53a;
R/Bioconductor v3.6.1; edgeR; DESeq; Limma; Seurat R package v3.1.0; Gene Set Enrichment Analysis (GSEA) v4.1.0
CHOPCHOP v3; SnapGene v5.3.2;
FACS Diva/v4.1 (Bectoin Dickinson); Flow Jo/v10 (Tree Star)
Image Lab v6.1 (Bio-Rad).

The code used in this paper for computational analyses is available upon a request.

For manuscripts utilizing custom algorithms or software that are central to the research but not yet described in published literature, software must be made available to editors and reviewers. We strongly encourage code deposition in a community repository (e.g. GitHub). See the Nature Portfolio guidelines for submitting code & software for further information.

Data

Policy information about availability of data

All manuscripts must include a data availability statement. This statement should provide the following information, where applicable:

- Accession codes, unique identifiers, or web links for publicly available datasets
- A description of any restrictions on data availability
- For clinical datasets or third party data, please ensure that the statement adheres to our policy

The RNA sequencing data reported in this paper have been deposited in the Sequence Read Archive (SRA, www.ncbi.nlm.nih.gov/sra/) under accession number PRJNA510181 and PRJNA983851. Reads containg sequence information were aligned onto hg38 for humans (https://www.ncbi.nlm.nih.gov/datasets/genome/GCF_000001405.26/) and macFas5 for primates (https://www.ncbi.nlm.nih.gov/datasets/genome/GCF_000364345.1/) genome references.

Research involving human participants, their data, or biological material

Policy information about studies with human participants or human data. See also policy information about sex, gender (identity/presentation), and sexual orientation and race, ethnicity and racism.

Reporting on sex and gender	N/A
Reporting on race, ethnicity, or other socially relevant groupings	N/A
Population characteristics	Human embryonic and fetal hearts (n=7) obtained from abortion materials between four and ten weeks of gestation from healthy subjects.
Recruitment	Embryonic/fetal materials collection was carried out at Karolinska University Hospital in Huddinge (Sweden). Only after the patient decided to undergo abortion for any reason, the medical staff at the Gynecology department informed her and her partner (or closest relatives) about the possibility to donate the embryo/fetus for only research purposes with documents describing the kinds of research that would be performed. After giving their informed consent for donation of the embryo or fetus, the patient underwent surgical abortion, and the aborted material was dissected under sterile conditions. As such, due to being entirely dependent on the patients' decisions in the collection of the embryonic or fetal hearts, there may be some sort of selection bias unavoidable, which may have some impact to results. The participants did not receive specific compensation.
Ethics oversight	Karolinska Institutet (Sweden) with the approved ethical permission number (Dnr 2015/1369-31/2).

Note that full information on the approval of the study protocol must also be provided in the manuscript.

Field-specific reporting

Please select the one below that is the best fit for your research. If you are not sure, read the appropriate sections before making your selection.

☒ Life sciences ☐ Behavioural & social sciences ☐ Ecological, evolutionary & environmental sciences

For a reference copy of the document with all sections, see nature.com/documents/nr-reporting-summary-flat.pdf

Life sciences study design

All studies must disclose on these points even when the disclosure is negative.

Sample size	Sample size was not pre-determined. Based on the high robustness of in vitro cardiac and vascular differentiation as well as in vivo kidney capsule experiments as described in the manuscript, we considered a sample size of at least n = 3 to be sufficient for experiments including statistical analysis.
Data exclusions	In the single-cell RNA-seq analysis, low expression genes (i.e., the number of expressed cells [RPKM>5] <5) and poor-quality cells (i.e., the number of expression genes [RPKM>1] <1500 or maximum correlation coefficient value <0.35) were filtered before further analysis.
Replication	The reproducibility of in vitro hESC differentiation and in vivo cardiac graft formation on murine kidney capsules with or without treatment with modified mRNA (or recombinant protein) of defined factors (e,g, PLGF) was validated in at least 3-5 biological replicates (e.g., independent in vitro differentiation), which showed robustness of the results. With the exception of single-cell RNA-seq analysis, the reproducibility of other experiments (i.e., ChIP, western blotting) was similarly validated in at least 3-5 biological replicates. All attempts for replication were successful.
Randomization	In vitro treatment groups (modified mRNA of each among 24 growth factors or control) were randomly assigned into the culture well and plates of WT hESCs. In vivo treatment groups (Intact/GFP-modRNA/PLGF-modRNA-heart progenitors) were also randomly assigned into immunocompromised mice. Randomization was not relevant to other experiments where no applicable comparisons were not conducted within the same batch of procedures.

Blinding

Investigators were not blinded to experimental groups. For the analyses of the FACS, ChIP, blotting and RNA-seq data blinding was not necessary because these analyses are observer-independent. For quantification of immunofluorescence images, blinding was not feasible since different conditions were detectable based on morphological appearances.

Reporting for specific materials, systems and methods

We require information from authors about some types of materials, experimental systems and methods used in many studies. Here, indicate whether each material, system or method listed is relevant to your study. If you are not sure if a list item applies to your research, read the appropriate section before selecting a response.

Materials & experimental systems

n/a	Involved in the study	
☐	☒	Antibodies
☐	☒	Eukaryotic cell lines
☒	☐	Palaeontology and archaeology
☐	☒	Animals and other organisms
☒	☐	Clinical data
☒	☐	Dual use research of concern
☒	☐	Plants

Methods

n/a	Involved in the study	
☒	☐	ChIP-seq
☐	☒	Flow cytometry
☒	☐	MRI-based neuroimaging

Antibodies

Antibodies used

Primary antibodies:
anti-alpha smooth muscle actin (SMA); Immunostaining, clone 1A4, Sigma-Aldrich, #A2547, 1:200
anti-beta actin-HRP; Western blotting, Cell Signaling Technology, #5125S, 1:2000
anti-CD34-APC; Flow cytometry, clone 581, BD Biosciences, #555824 1:100
anti-EOMES; ChIP, clone EPR21950-241, Abcam, #ab216870, 1:50
anti-EOMES; Western blotting, polyclonal, Abcam, #ab23345, 1:1000
anti-ISL1; Immunostaining, clone 39.4D5, DSHB, #39.4D5, 1:20
anti-ISL1-PE; Flow cytometry, clone Q11-465, BD Biosciences, #562547, 1:100
anti-Ki67; Immunostaining, clone B56, BD Bioscience, #550609, 1:25
anti-Ki67-FITC; Flow cytometry, clone B56, BD Biosciences, #556026, 1:20
anti-MF20; Immunostaining, DSHB, #P13538, 1:10
anti-MLC2V; Immunostaining, polyclonal, Proteintech, #10906-1-AP, 1:100
anti-PDGFRB-PE; Flow cytometry, clone 28D4, BD Biosciences, #558821, 1:100
anti-PECAM1; Immunostaining, clone 89C2, Cell Signaling Technology, #3528, 1:50
anti-PECAM1-AF647; Flow cytometry, clone M89D3, BD Biosciences, #558094, 1:100
anti-PLGF; Immunostaining&western blotting; polyclonal, Abcam; #ab9542; 1:100 (immunostaining) & 1:500 (western)
anti-SM22 Flow cytometry&immunostaining; polyclonal, Abcam, #ab10135, 1:100
anti-SOX17; Western blotting&ChIP, clone EPR20684, Abcam, #ab224637, 1:500 (western) & 1:30 (ChIP)
anti-TNNT2; Immunostaining, clone 13-11, Thermo Fisher Scientific, #MS-295-P1, 1:50
anti-TNNT2; Immunostaining, clone EPR3695, Abcam, #ab91605, 1:50
anti-TNNT2-APC; Flow cytometry, clone REA400, Miltenyi Biotec, #130-120-403, 1:100
anti-VE-cadherin; Immunostaining, polyclonal, R&D, #AF938, 1:50
anti-VE-cadherin-PE; Flow cytometry, clone 55-7H1, BD Biosciences, #560410, 1:100
anti-Vimentin; Immunostaining, polyclonal, Millipore, #AB5733, 1:1000

Secondary antibodies:
Donkey anti-rabbit Alexa Fluor 488, Invitrogen, #A21206
Donkey anti-goat Alexa Fluor 594, Invitrogen, #A11058
Donkey anti-mouse Alexa Fluor 647, Invitrogen, #A31571
Goat anti-mouse IgG2a Alexa Fluor 568, Invitrogen, #A21134
Goat anti-mouse IgG1 Alexa Fluor 647, Invitrogen, #A21240
Donkey anti-mouse Alexa Fluor 488, Invitrogen, #A21202
Donkey anti-rabbit Alexa Fluor 647, Invitrogen, #A31573
Donkey anti-mouse Alexa Fluor 555, Invitrogen, #A31570
Goat anti-chicken Alex Fluor 647, Invitrogen #A21449
Goat anti-rabbit Alexa Fluor 488, Invitrogen, #A11008
Goat anti-mouse IgG2b Alexa Fluor 555, Invitrogen, #A21147

Isotype control:
REA Control Antibody, human IgG1-APC (for anti-TNNT2-APC #130-120-403); clone REA293; Miltenyi Biotec, #130-120-709, 1:100
Mouse IgG1-PE (for anti-ISL1-PE #562547 and anti- VE-cadherin-PE #560410); clone MOPC-21, BD Biosciences, #554680, 1:50
Mouse IgG1-FITC (for anti-Ki67-FITC #556026); clone MOPC-21, BD Biosciences, #556026, 1:20
Mouse IgG2a-AF647 (for anti-PECAM1-AF647 #558094); clone G155-178, BD Biosciences, #557715, 1:100
Mouse IgG2a-PE (for anti-PDGFRB-PE #558821); clone MOPC-173, BD Biosciences, #565363, 1:100

Horse anti-mouse IgG conjugated with HRP, Cell Signaling Technology, #7076s

	Goat anti-rabbit IgG conjugated with HRP, Cell Signaling Technology, #7074s
Validation	All primary antibodies were validated for immunocytochemistry/immunohistochemistry/western blotting/chromatin immunoprecipitation analysis of human samples by the respective manufacturers. All secondary antibodies were validated for immunocytochemistry/immunohistochemistry/western blotting analysis by the respective manufacturers.

Eukaryotic cell lines

Policy information about cell lines and Sex and Gender in Research

Cell line source(s)	The human ESC line WA09 (H9) was purchased from WiCell Research Institute (USA).
Authentication	The pluripotency and karyotype of hESC lines including genetically modified lines were validated based on typical colony morphology, expression of pluripotency markers (e.g., POU5F1) assessed by FACS, and standard G-band karyotype analysis. Trilineage potential was assessed by FACS analysis of specific markers of the three germ layers following trilineage differentiation in embryoid bodies or monolayers.
Mycoplasma contamination	All cell lines used tested negative for mycoplasma.
Commonly misidentified lines (See ICLAC register)	No commonly misidentified lines were used.

Animals and other research organisms

Policy information about studies involving animals; ARRIVE guidelines recommended for reporting animal research, and Sex and Gender in Research

Laboratory animals	Non-human primates (macaque fascicularis; female, 5-8 yrs old) and immunodeficient NOD.Cg-PrkdcSCID Il2rgtm1Wjl/SzJ (NSG) mice (male, 10-12 weeks old; Charles River). The mice were housed with normal diets under conditions, including 12h dark/light cycle, ambient temperature (20-22 °C), and humidity (40-60%).
Wild animals	No wild animals were used in the study.
Reporting on sex	Male NOD.Cg-PrkdcSCID Il2rgtm1Wjl/SzJ (NSG) mice were used for the characterization of the human-mouse chimeric heart muscle grafts that were generated by transplantation of hESCs into murine kidney capsules.
Field-collected samples	No field collected samples were used in the study.
Ethics oversight	The study was performed in accordance with the Declaration of Helsinki and the guidelines from Directive 2010/63/EU, and all the protocols in the animal (primates and mice) works were approved by the institutional review board at Karolinska Institutet (KI) with ethical permission numbers (N277/14 and N227-14).

Note that full information on the approval of the study protocol must also be provided in the manuscript.

Flow Cytometry

Plots

Confirm that:

☒ The axis labels state the marker and fluorochrome used (e.g. CD4-FITC).

☒ The axis scales are clearly visible. Include numbers along axes only for bottom left plot of group (a 'group' is an analysis of identical markers).

☒ All plots are contour plots with outliers or pseudocolor plots.

☒ A numerical value for number of cells or percentage (with statistics) is provided.

Methodology

Sample preparation	- The harvested embryonic hearts were micro-dissected into one whole heart or 4 compartments (OFT, RV, LV, and atria), and the divided heart regions were cut into small pieces and dissociated into single cells by incubation with collagenase. After staining the dissociated cells with DAPI (Thermo Fisher Scientific), DAPI-negative (live) single cells were sorted into 384-well plates containing cell lysis buffer, customized for the Smart-seq2 approach, using a fluorescence-activated cell sorter (FACSARIA III; BD Biosciences).<br>- In vitro cells were dissociated into single cells with Accutase for 5-10 min, washed in PBS, and blocked for 30 min in fluorescence activated cell sorting (FACS) buffer (1% bovine serum albumin and 10% horse serum in PBS) at 4 degrees C. Staining for cell surface antigens was first performed in the vasculogenesis assay of CM differentiation and in SMC and EC differentiation for 30 min at 4 degrees C using the primary antibodies. Cells were then fixed with 4% paraformaldehyde, permeabilized, blocked, and stained for intracellular antigens for 30 min at room temperature using the primary antibodies, followed by staining with an Alexa-Fluor 647-conjugated secondary antibody (BD Biosciences) for 15 min at 4 degrees C.

Instrument	A flow cytometer FACSARIA III (Beckton Dickinson)
Software	FACS Diva/v4.1 (Beckton Dickinson) and FlowJo/v10 (Tree Star)
Cell population abundance	- In the embryonic heart study, DAPI-negative cells occupied >95-98%.<br>- In vitro WT hESC-CM differentiation: (day 6) %Ki67+: 50%, %ISL1+: 90%, %TNNT2+ 20-30%; (day 15) %Ki67+: 10%, %ISL1+ 50%, %TNNT2+: 75%<br>- in vitro WT hESC-SMC differentiation: (day 6) %PDGFRB+ 75%<br>- in vitro WT hESC-EC differentiation: (day 6) %VEC+ 50%
Gating strategy	Analyze and sort the stained cells using a flow cytometer and cell sorter (e.g., FACSARIA III [BD Biosciences]) equipped with a 100 mm nozzle at 4C temperature. Keep the flow rate between 800–1,200 events per second.<br>a. Use the unstained and single color-stained controls to set appropriate PMT voltages and adjust compensation using a cell analyzing software (e.g., FACS Diva [BD Biosciences]).<br>b. Gate single cells with the FSC-A versus SSC-A plot, the FSC-W versus FSC-H plot, and the SSC-W versus SSC-H plot to discard cell debris and doublets (Figure 2A). When performing live cell sorting, gate DAPI- or PI-negative cell populations (see step 12a Note).<br>c. Create appropriate sorting gates for the samples through concurrently analyzing and referring to the isotype antibody-stained and the marker-positive or negative cell-type controls.

☒ Tick this box to confirm that a figure exemplifying the gating strategy is provided in the Supplementary Information.
